# The Effect of Retention Time and Seasonal Variation on the Characterization of Phyto-Remediated Aquaculture Wastewater in a Constructed Wetland

**DOI:** 10.3390/biology14101390

**Published:** 2025-10-12

**Authors:** Shadrach A. Akadiri, Pius O. O. Dada, Adekunle A. Badejo, Olayemi J. Adeosun, Akinwale T. Ogunrinde, Oluwaseun T. Faloye, Viroon Kamchoom, Oluwafemi E. Adeyeri

**Affiliations:** 1 Department of Agricultural and Bioresources Engineering, Federal University of Agriculture, Abeokuta 111101, Ogun State, Nigeria; 2 Department of Agriculture and Natural Resources, Ondo State Local Government Service Commission, Akure 340110, Ondo State, Nigeria; 3 Department of Civil Engineering, Federal University of Agriculture, Abeokuta 111101, Ogun State, Nigeria; 4 Key Laboratory of Ecological Safety and Sustainable Development in Arid Lands, Northwest Institute of Eco-Environment and Resources, Chinese Academy of Sciences, Lanzhou 730070, China; ogunrindeakinwale@gmail.com; 5 Department of Water Resources Management and Agrometeorology, Federal University Oye, Oye 370112, Ekiti, Nigeria; 6 Department of Civil Engineering, Landmark University, Omu Aran 251103, Kwara State, Nigeria; 7 Excellent Centre for Green and Sustainable Infrastructure, School of Engineering, King Mongkut’s Institute of Technology Ladkrabang, Bangkok 10520, Thailand; 8 ARC Centre of Excellence for the Weather of the 21st Century, Fenner School of Environment and Society, The Australian National University, Australian Capital Territory, Canberra 2600, Australia

**Keywords:** phytoremediation, vertical sub-surface flow system, *Phragmites karka*, *Typha latifolia*, heavy metal, physico-chemical characterization

## Abstract

**Simple Summary:**

This study investigates the effectiveness and efficiency of macrophyte plants (*Phragmites karka* and *Typha latifolia* in removing heavy metals and some nutrients from aquaculture wastewater in a constructed wetland. Raw and treated wastewater from constructed wetlands were collected and analyzed using standard laboratory procedures for the determination of physicochemical properties and heavy metals. The performance of the wetland was assessed based on the type of plant, retention time, and season. The results revealed a significant impact of plant type, retention time, and season on the removal of nutrients and heavy metals, when compared to the raw wastewater’s physicochemical and heavy metal values. These findings suggest the need to adopt a phytoremediation strategy using *Phragmites karka* and *Typha latifolia* to promote the reuse of aquaculture wastewater for further agricultural purposes in areas where water is limited.

**Abstract:**

The insufficient availability of safe water has emerged as a prevalent issue severely impacting public health in developing nations. Moreover, studies reporting the efficacy of treatment plants (TPs)—specifically *Phragmites karka* and *Typha latifolia*—in removing toxic elements in aquaculture wastewater are scanty. Therefore, this study is aimed at investigating the effects of hydraulic retention time (HRT), seasonal variations, and TPs on the removal efficiency of pollutants from a vertical subsurface flow constructed wetland (VSSF-CW) in Nigeria. The experiments spanned three seasons (November–December–January—NDJ; March–April–May—MAM; and July–August–September—JAS) of the year, with samples collected from the CW at 7 day intervals for analysis. The aquaculture wastewater was analyzed in the laboratory to determine its chemical and toxic compositions before and after the introduction of treatment plants. Three-way ANOVA was used to analyze the main and interactive effects between HRT, seasons, and TPs on the physicochemical properties of the CW’s effluents. The removal efficiency was determined to evaluate the performance of the constructed wetland in comparison to the treatment plants. Results showed that these constructed wetlands effectively removed contaminants, with significant differences (*p* < 0.05) mostly observed in the effects of treatment plant types and seasons on the chemical and heavy metal concentrations. This was further confirmed by the main effects of HRT, seasons, and treatment plant choice, which significantly (*p* < 0.05) influenced treatment efficiency. Removal efficiencies increased with longer HRTs, reaching peak removal efficiencies of approximately 69, 67, and 61% for Na, K, and Ca, respectively. The BOD and COD reached 85 and 90% removal efficiency, while removal efficiency of 100% was achieved for most heavy metals at 21 day retention time. In summary, the study found that TPs (*Phragmites karka* and *Typha latifolia*), HRT, and seasonal variation are important for treating integrated poultry and aquaculture wastewater in a VSSF CWs.

## 1. Introduction

Wetlands are unique ecosystems that offer manifold environmental and economic benefits, serving as habitats for aquatic life and wildlife, including rare and endangered species [1]. They play a vital role in wastewater purification, flood mitigation, and coastal erosion prevention. Wetlands also provide sustainable, cost-effective resources for human use and present opportunities for academic study and recreational activities [1]. Furthermore, it facilitates a smooth transition between arid regions and aquatic environments. In numerous underdeveloped and developing nations, the available wastewater treatment methods lag significantly behind those employed in developed countries. In Nigeria, for instance, over 50% of the population resides in rural areas where surface water bodies constitute the primary source for both social and domestic water needs [2]. Unfortunately, agricultural runoff and poorly planned sewage systems are major contributors of chemical and microbiological contaminants into these surface waters, posing a severe threat to the health and livelihoods of rural communities. This environmental challenge can be attributed to a lack of expertise in maintaining conventional treatment systems and economic limitations [3,4]. Despite the introduction of regulations regarding wastewater discharge conditions in 1970, the Nigerian Environmental Agency adopted wastewater treatment techniques that proved unsustainable due to their high maintenance costs [5]. Consequently, there is an urgent demand for the development of a sustainable and economically feasible wastewater treatment approach.

In addition to being expensive, conventional wastewater treatment methods are challenging to maintain, and the resulting sludge requires advanced treatment before disposal. Recognizing this issue, engineers and environmentalists have developed a system that emulates natural wastewater treatment processes. Constructed wetlands (CWs) are the primary outcome of this innovation, typically categorized into two types: free water surface (FWS) and subsurface flow (SSF). The FWS type consists of parallel containers or channels with impermeable soil bases, featuring the cultivation of aquatic plants and shallow water depths, typically ranging from 0.1 to 0.6 m [6]. On the other hand, SSF CWs are designed to be more effective compared to FWS, serving as advanced treatment systems as well. They comprise channels with relatively impermeable bases filled with substrate media that support the growth of macrophytes. Literature indicates that CW treatment units have been successfully employed for treating various types of wastewater, including but not limited to domestic, agricultural, landfill leachate, chemical, and petrochemical industry effluents. This is due to their advantages such as minimal sludge generation, low energy requirements, simplicity of operation, and low maintenance demands [7,8].

The processes involved in eliminating pollutants within a CW can be categorized into several methods: physical processes such as accumulation and filtration, chemical processes like adsorption and precipitation, and biological processes including microbial breakdown, plant adsorption, and natural decay. CWs employ aquatic macrophytes, commonly found in natural environments, for wastewater treatment from various sources. Some of these macrophytes include Vetiver grass, *V. nigritana*, *P. Australis*, *Phragmites karka*, and *Typha latifolia*. Vetiver grass is a tall, rapidly growing tropical plant renowned for its extensive and advanced root system, capable of penetrating deep into soil layers [9,10]. *V. nigritana* demonstrates remarkable adaptability to high levels of salinity, acidity, alkalinity, and a wide array of heavy metals [11]. *P. Australis* has also been noted for its valuable ecosystem service in reducing heavy metal pollutants [12]. Both *Phragmites karka* and *Typha latifolia* are considered significant wetland plants due to their ability to flourish in aquatic environments, their ecological contributions, and their roles in supporting diverse plant and animal communities, as well as their effectiveness in removing heavy metals from wastewater [10]. Moreso, the above-stated macrophytes are well-known for their roots, including *Hyacinthus orientalis*, *Lemna genus*, and *Populus genus*. Their complex root structures serve as natural filters [13] for suspended contaminants, providing a wealth of surfaces for microbial colonization. Meanwhile, the antibacterial qualities of exudates combat pathogenic and fecal indicator bacteria [8,10]. Aquaculture, residential, sewage, and industrial wastewaters have all been remedied using the above-stated plants. Notably, there is currently a lack of research investigating the application of CWs planted with *Typha latifolia* and *Phragmites karka* in the treatment of combined effluent from aquaculture and poultry. In addition, studies that comprehensively investigated the combined effects of treatment plants, retention time, and seasons in a constructed wetland are scant to date [14,15].

In constructed wetlands (CWs), several crucial parameters come into play, including retention time, loading rates (both hydraulic and organic), plant varieties, and the choice of substrate and filter media, all of which can significantly enhance the quality of the effluents discharged from CW outlets [1,16]. Retention time stands out as a particularly critical parameter to consider. However, a noticeable gap remains in research regarding the treatment of poultry and aquaculture wastewater using Vertical Subsurface Flow Constructed Wetlands (VSSF-CWs), particularly in terms of the intricacies of design parameters and the impact of seasonal variations.

In contrast to conventional treatment systems, CWs present an eco-friendly approach that is cost-effective, demands fewer operational and maintenance resources, fosters a habitat for wetland organisms, can adapt to fluctuations in water flow, promotes wastewater reuse and recycling, and holds great potential for application in developing countries, especially within small rural communities. Nevertheless, achieving sustainable management and the successful application of these systems continues to pose challenges [17].

Throughout history, Constructed Wetlands (CWs) have witnessed rapid development and widespread adoption in regions such as North America, Australia, and Europe. However, in Asia and Africa, they are increasingly recognized as a valuable alternative to conventional treatment methods. One of the notable advantages of CWs is their adaptability to local conditions, utilizing readily available materials and local labour, which makes them particularly suitable for underdeveloped and developing countries.

This study focuses on assessing the capacity of CWs for treating agricultural wastewater in Nigeria, emphasizing their effectiveness and performance within a local context. Consequently, the primary objective of this research is to investigate how variations in hydraulic retention time (HRT) impact the removal efficiency of Vertical Subsurface Flow Constructed Wetlands (VSSF-CW) when applied to rural wastewater treatment throughout different seasons. The key aims are twofold: (a) to assess the influence of operational parameters such as hydraulic retention time and (b) to investigate the seasonal effects on pollutant removal efficiency. In pursuit of these goals, this study evaluates the performance of a pilot-scale wetland under varying hydraulic retention times and examines the efficiency of two specific macrophyte species, *Phragmites karka* and *Typha latifolia*, in treating a combination of aquaculture and poultry wastewater.

## 2. Methodology

### 2.1. The Study Location and the Description of the Experimental Setup

The research was carried out at the experimental farm of the Centre of Excellence in Agriculture Development and Sustainable Environment (CEADESE), located at the Federal University of Agriculture in Abeokuta, Ogun State, Nigeria. This site is positioned at approximately 7°9′ north latitude and 3°21′ east longitude. The study area falls within the tropical rainforest climatic zone of West Africa. It is characterized by temperatures ranging from around 12.5 °C to slightly above 30.5 °C, with an average temperature of 22 °C. The region experiences two main seasons, namely the wet and dry seasons, which are determined by the area’s rainfall patterns. The average annual rainfall exceeds 1500 mm and follows a bi-modal pattern. The wet season typically begins in early April and lasts until early October or November, while the dry season covers the remaining months.

**Figure 1:**
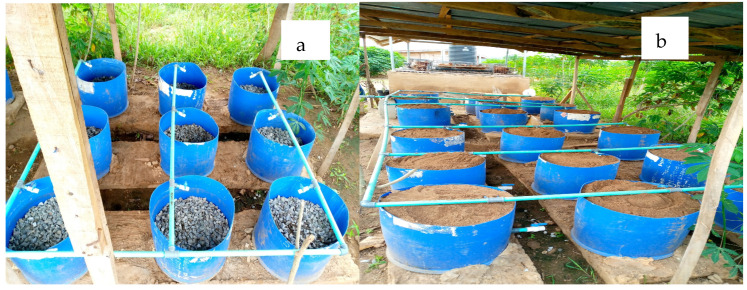
Vertical subsurface wetland filled with coarse gravel (**a**) and coarse gravel + sand (**b**).

**Figure 2:**
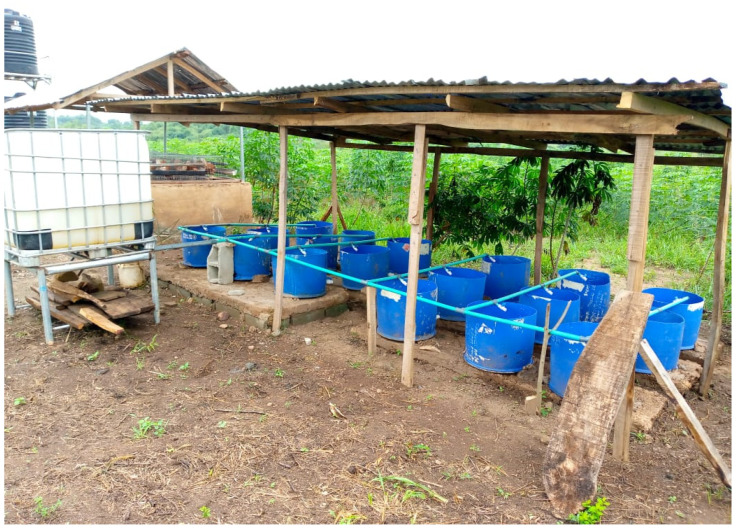
Setup of vertical subsurface constructed wetlands used to treat wastewater under the conditions of seasonal variation, treatment type, and retention time.

The Vertical Subsurface Flow Constructed Wetland (VSSF-CW) was specifically engineered to treat the effluent originating from an integrated poultry and aquaculture wastewater system. These cylindrical CW units have dimensions of 0.6 m in diameter and 0.5 m in height, providing a total volume of 105 L. The chosen filter media consisted of a mixture of gravel and coarse sand. The cylindrical containers were filled with a layer of 20 cm thickness for both gravel and sand. The gravels were at the bottom part, while the coarse sand was at the upper part, as illustrated in Figure 1a,b. The design principles considered encompassed factors such as flow rate, the aspect ratio (length-to-width ratio), hydraulic retention time, and hydraulic loading rate, drawing from the insights of [18,19,20]. For the treatment of the combined poultry and aquaculture wastewater, the VSSF-CWs were thoughtfully planted with two locally available plant species, *Phragmites karka* and *Typha latifolia*. The *Phragmites karka* was established using asexual propagation techniques such as stolons and rhizomes, while *Typha latifolia* was established with cuttings obtained from mature plants collected from a nearby stream. Each planting medium comprised nine replications, with a density of nine plants per cylindrical container (32 plants/m^2^), as inspired by the work of Chang et al. [21]. Wastewater was allowed to enter the constructed wetland by downflow as illustrated in Figure 2. The right side of the experimental unit was planted with *Typha latifolia*, while the left side was planted with *Phragmites karka*.

To maintain subsurface flow conditions and prevent issues like mosquito breeding and odours, the water level within the CWs was consistently maintained up to the second (sand) layer. The experiments were systematically conducted on a seasonal basis, spanning intervals of three months to encompass the major climatic seasons prevalent in Nigeria. Wastewater samples were diligently collected using pre-acid-washed plastic bottles, temporarily stored in ice coolers, and subsequently transported to the laboratory for analytical assessments.

### 2.2. Operation of the Experiment

The study was conducted over a span of time, commencing in November 2021 and concluding in September 2022. During this timeframe, a series of three consecutive experiments was undertaken, corresponding to the seasons of November–December–January (NDJ), March–April–May (MAM), and July–August–September (JAS). After the establishment of the macrophyte, which had fully grown by 3 months, each experimental run was allowed to extend for 21 days. Within this period, samples were systematically collected from each VSSF-CW at 7 day intervals. In each season, retention periods of 7, 14, and 21 days were maintained, with control valves connected to the CWs closed, and they were opened when the exact retention periods were reached. The operation of the CWs was such that the retention periods of 7 days were completed before commencing the experiment for 14 day retention period, and finally the 21 day retention period. The inflow of effluent into the CWs was within an hour. These samples were meticulously tested to evaluate the pollutant levels present in the treated wastewater. To ensure that the VSSF-CW units adapted to the new hydraulic retention time of 7 days, a one-month acclimatization period was instituted before performance data retrieval. The process of wastewater sampling and subsequent analysis involved preliminary characterization, distinguishing between wet and dry seasons, through the application of a grab sampling technique. The water samples were gathered and placed in pristine, see-through 75cl bottles. The samples were stored at 4 °C until transportation to the laboratory. Over the course of four weeks, nine replicates of water samples were gathered weekly from designated sampling points on the VSSF-CW units. All collected samples underwent comprehensive analysis, encompassing physical properties (temperature and turbidity) and chemical properties (pH, acidity, total alkalinity [TA], biological oxygen demand [BOD_5_], chemical oxygen demand [COD], total nitrogen [TN], chlorides [Cl^−^], nitrate [NO_3_^−^], sulphates [SO_4_^2−^], sodium [Na], potassium [K], calcium [Ca], magnesium [Mg], arsenic [As], cadmium [Cd], copper [Cu], chromium [Cr], cobalt [Co], iron [Fe], lead [Pb], manganese [Mn], zinc [Zn], and nickel [Ni]. All sample retrieval and handling procedures adhered to standardized protocols [22]. The specific test procedures for each parameter are listed in Table 1. The treatment efficiency of the CWs was calculated based on the removal of pollutants for the parameters analyzed using Equation (1).

(1) $$\%\;R=\left(\frac{P_{i}-P_{e}}{P_{i}}\right)\times 100$$

where % *R* stands for the percentage of pollutant removal in the VSSF-CW; $P_{i}$ and $P_{e}$ stands for the concentration of pollutants in the influent and effluent, respectively, in mg/L.

### 2.3. Statistics

The experimental results underwent rigorous statistical analysis, employing Minitab version 17 and Microsoft Excel 2016 software. Descriptive analysis was employed to provide a concise overview of the fundamental characteristics of the data within the study. This entailed generating straightforward summaries, including the calculation of means and standard deviations for the physical, chemical, and microbiological parameters. To assess the variations in removal efficiency across seasons, treatment plants, and hydraulic retention times, a comprehensive analysis was conducted. This involved the utilization of multiple analysis of variance (ANOVA) tests, including one-way and three-way tests. The one-way ANOVA enabled the separation of means among the treatment plants and the raw aquaculture wastewater, while the three-way ANOVA enabled the determination of the main and interactive effects between seasons, retention time, and treatment plants on the wastewater’s physicochemical properties and heavy metal content. Subsequently, post hoc analysis was carried out using Tukey’s test, executed at a significance level of 95%. A *t*-test was used to separate the means of the removal efficiency between the two treatment plants, and at the different seasons and retention times.

## 3. Results and Discussion

**Figure 3:**
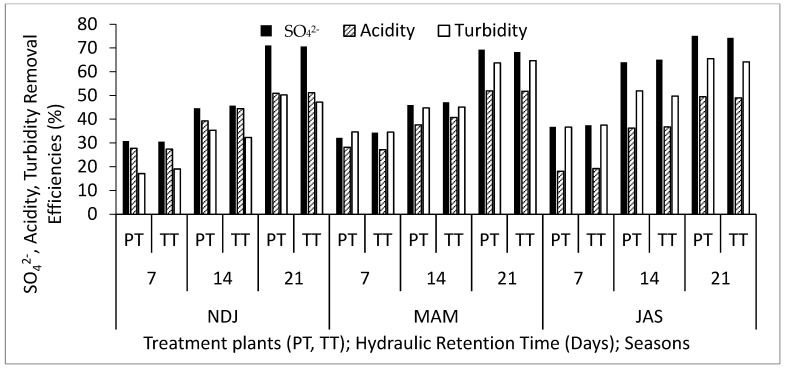
Removal efficiencies of SO_4_^2−^, acidity, and turbidity for different treatment plants, hydraulic retention time, and seasons in the VSSF CW treatment system. **Note:** PT stands for *Phragmites karka*, TT stands for *Typha latifola*, NDJ—November, December, and January, MAM—March, April, and May, JAS—July, August, and September.

**Figure 4:**
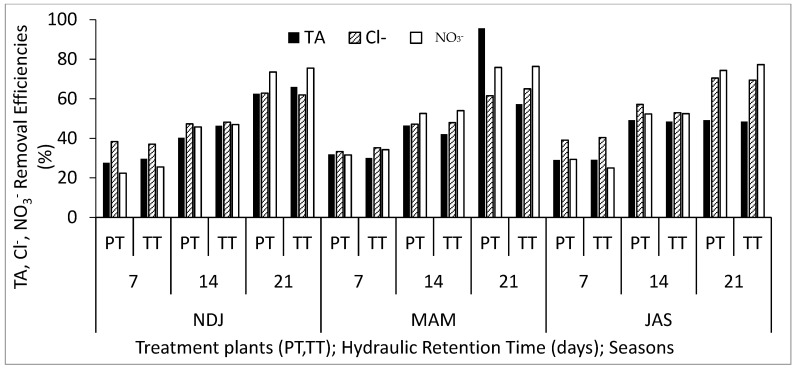
Removal efficiencies of TA, Cl^−^, and NO_3_^−^ for different treatment plants, hydraulic retention time, and seasons in the VSSF CW treatment system. **Note:** PT stands for *Phragmites karka*, TT stands for *Typha latifola*, NDJ—November, December, and January, MAM—March, April, and May, JAS—July, August, and September.

**Figure 5:**
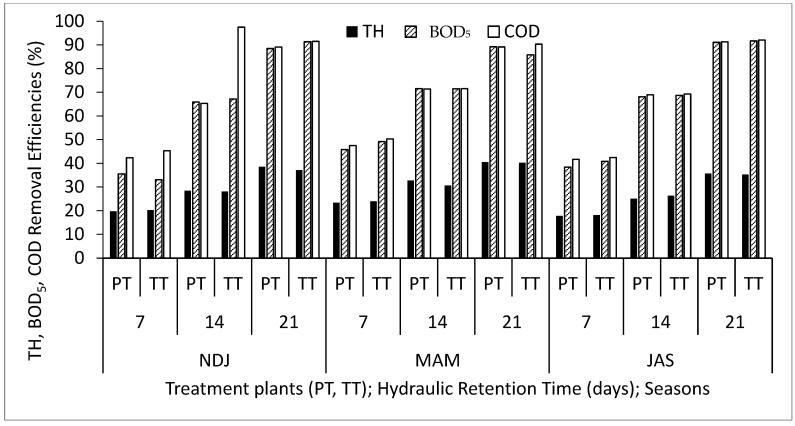
Removal efficiencies of TH, BOD_5_, and COD for different treatment plants, hydraulic retention time, and seasons in the VSSF CW treatment system. **Note:** PT stands for *Phragmites karka*, TT stands for *Typha latifola*, NDJ—November, December, and January, MAM—March, April, and May, JAS—July, August, and September.

**Figure 6:**
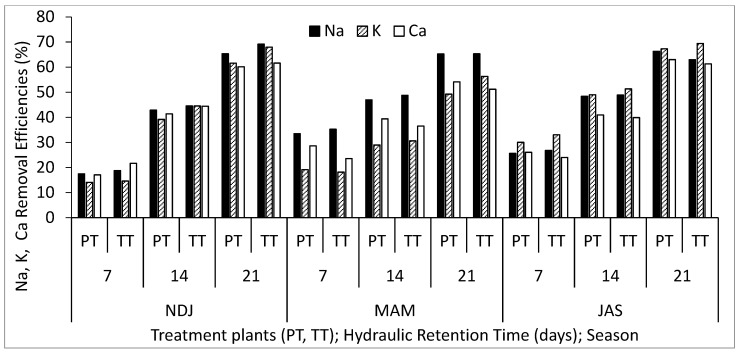
Removal efficiencies of Na, K, and Ca for different treatment plants, hydraulic retention time, and seasons in the VSSF CW treatment system. **Note:** PT stands for *Phragmites karka*, TT stands for *Typha latifola*, NDJ—November, December, and January, MAM—March, April, and May, JAS—July, August, and September.

**Figure 7:**
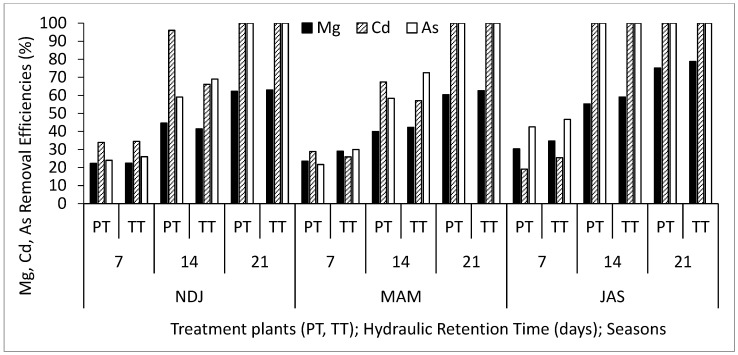
Removal efficiencies of Mg, Cd, and As for different treatment plants, hydraulic retention time, and seasons in the VSSF CW treatment system. **Note:** PT stands for *Phragmites karka*, TT stands for *Typha latifola*, NDJ—November, December, and January, MAM—March, April, and May, JAS—July, August, and September.

**Figure 8:**
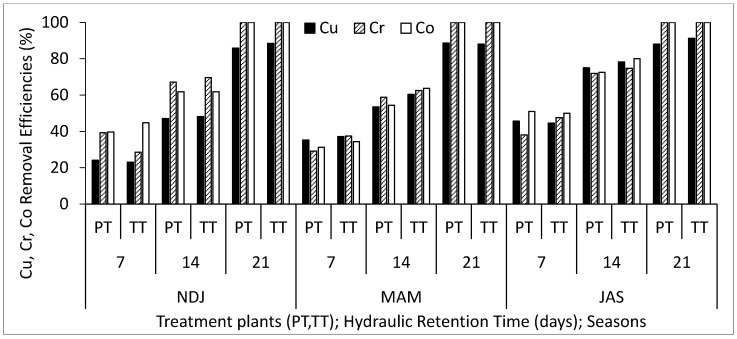
Removal efficiencies of Cu, Cr, and Co for different treatment plants, hydraulic retention time, and seasons in the VSSF CW treatment system. **Note:** PT stands for *Phragmites karka*, TT stands for *Typha latifola*, NDJ—November, December, and January, MAM—March, April, and May, JAS—July, August, and September.

**Figure 9:**
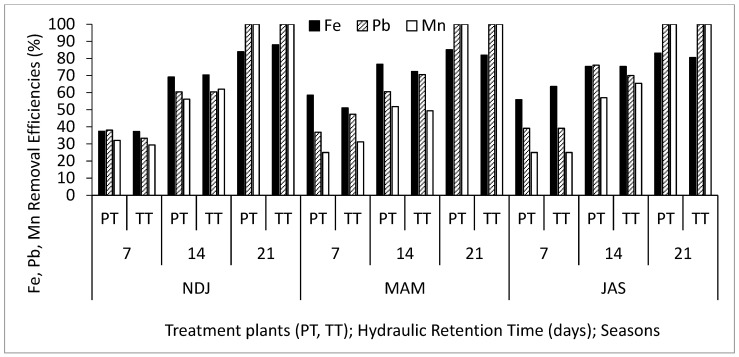
Removal efficiencies of Fe, Pb, and Mn for different treatment plants, hydraulic retention time, and seasons in the VSSF CW treatment system. **Note:** PT stands for *Phragmites karka*, TT stands for *Typha latifola*, NDJ—November, December, and January, MAM—March, April, and May, JAS—July, August, and September.

**Figure 10:**
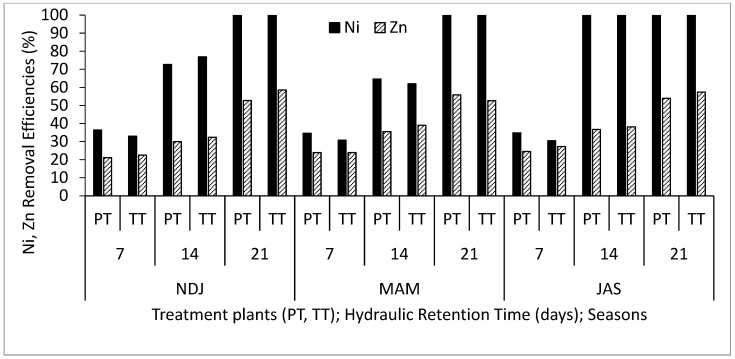
Removal efficiencies of Ni and Zn for different treatment plants, hydraulic retention time, and seasons in the VSSF CW treatment system. **Note:** PT stands for *Phragmites karka*, TT stands for *Typha latifola*, NDJ—November, December, and January, MAM—March, April, and May, JAS—July, August, and September.

Table 2 displays the average and standard deviation of physical and chemical properties concentrations in wastewater for the three periods (NDJ, MAM, and JAS) considered in the study. The values of the physicochemical properties in each period (NDJ, MAM, and JAS) are seen in supplementary data (Tables S1–S3). It offers a comparison between raw wastewater (RWW) and treated wastewater at hydraulic retention times (HRT) of 7, 14, and 21 days in Vertical Subsurface Flow Constructed Wetlands (VSSF-CWs) planted with *Phragmites karka* and *Typha latifolia* across all three seasons. Throughout the study, in situ observations were made for parameters such as pH, dissolved oxygen, colour, and temperature within the VSSF-CW. Additionally, monthly average air temperatures at a height of 2 m above the ground surface were summarized in Table 3. Notably, January marked the lowest temperature at 19.54 °C, while February recorded the highest at 31.13 °C. These months correspond to the dry season, characterized by the harmattan phenomenon. Importantly, these recorded temperatures consistently fell within the optimal operational range for CWs (20–35 °C) as recommended by [23,24]. This data’s variability highlights the study area’s favourable climate, supporting year-round growth and development of the plants within the CW [25]. Figure 3, Figure 4, Figure 5, Figure 6, Figure 7, Figure 8, Figure 9 and Figure 10 depict the removal efficiencies of turbidity, acidity, total alkalinity (TA), chloride (Cl^−)^, nitrate (NO_3_^−^), sulphate (SO_4_^2−^), chemical oxygen demand (COD), biological oxygen demand (BOD5), as well as the concentrations of sodium (Na), potassium (K), calcium (Ca), magnesium (Mg), arsenic (As), cadmium (Cd), copper (Cu), chromium (Cr), cobalt (Co), iron (Fe), lead (Pb), manganese (Mn), zinc (Zn), and nickel (Ni) across the three seasons throughout the study period. Furthermore, Table 4 and Table 5 present the primary and interactive effects of Hydraulic Retention Time (HRT), treatment plants, and seasons on the tested parameters.

### 3.1. Removal Efficiency of Parameters, Metals, and Heavy Metals

Figure 3, Figure 4, Figure 5, Figure 6, Figure 7, Figure 8, Figure 9 and Figure 10 present comprehensive data on the removal efficiencies of various physicochemical, metal, and heavy metal parameters in integrated poultry and aquaculture wastewater. These measurements were taken at different Hydraulic Retention Times (HRTs) of 7, 14, and 21 days. The study considers two treatment plants (PT—*Phragmites karka*; TT—*Typha latifolia*) and three distinct seasons (NDJ, MAM, and JAS). The constructed wetlands showcased impressive performance, with contaminants being consistently and significantly reduced within the initial 7 days of HRT, and these favourable trends continued through the 14 and 21 days of HRT. Notably, there were no discernible physical distinctions in the removal efficiencies between PT and TT planted CWs, nor did they significantly differ across the three seasons (NDJ, MAM, and JAS).

Figure 3 provides insights into the removal efficiencies of SO_4_^2−^, acidity, and turbidity. The recorded removal rates for SO_4_^2−^ at 7- 14, and 21 days HRT ranged from 30 to 76% across both treatment plants and seasons. These results highlight the remarkable sulphate and sulphur-related removal capabilities of the CW microcosms used in the study. Several factors may contribute to this, including the emission of hydrogen sulphide, absorption through plant uptake, and the deposition of sulphur compounds such as calcium sulphate, metal sulphide, and elemental sulphur [26,27]. Hou et al. [28] also emphasized the role of sulphur compound deposition on iron-rich gravel in CWs as a primary mechanism for SO_4_^2−^ removal. The reduction in acid content, ranging between 36 and 66% from 7 to 21 days of HRT under TT and PT treatment plants across the three seasonal periods, further underscores the CWs’ efficiency in removing acid-related chemicals. This aligns with findings from [29], who reported approximately 50% removal efficiencies for certain pharmaceutical products in CWs and noted statistical differences in the removal of specific compounds, emphasizing the need for integrated CW designs. Turbidity, responsible for water sample clarity due to solid substances, decreased by 17 to 66% over the 21 day HRT period. This reduction can be attributed to the effective filtration system in sub-surface CWs. Additionally, the role of macrophytes, possibly in collaboration with microorganisms, in turbidity reduction deserves acknowledgment.

Figure 4 demonstrates the removal efficiencies for TA, Cl^−^, and NO_3_^−^. The reduction in TA concentration levels, ranging from 28% to approximately 95%, within the 21-day HRT was substantial and significantly contributed to the reduction in Ammonia and Nitrogen content in the wastewater. The Cl^−^ concentration removal efficiency varied from 32 to 70%. Consistent with the present study, Schück and Greger [30] conducted research on 34 wetland plants and identified certain large biomass plants, such as *C. riparia* and *P. arundinacea*, as having high tolerance and accumulation capacity for Cl^−^ phytodesalination. The peak removal efficiency (76%) for NO_3_^−^ occurred during the MAM season after 21 days HRT, with the CW’s substrate media playing a crucial role in removing excess NO_3_^−^. Zeng et al. [31] discovered a promising substrate medium, such as palygorskite self-assembled composite material (PSM) for nitrogen compound removal in CWs.

Figure 5 provides insights into the removal efficiencies of TH, BOD_5_, and COD at different HRTs, treatment plants, and seasons. The data displayed no significant variation in removal efficiencies among different HRTs, treatment plants, and climatic seasons within the study area. TH removal, ranging from 18 to 40% within 21 days of HRT, suggests that CW treatment can effectively reduce salt content that may otherwise impact crop growth and cause material corrosion. Moreover, higher removal efficiency for COD (90%) compared to BOD_5_ (85%) after 21 days of HRT is noteworthy. This high removal efficiency for both parameters can be attributed to the combined effects of substrate media, microorganisms, and wetland plants, consistent with previous studies [7,8]. Figure 6 illustrates the removal efficiencies of Na, K, and Ca at different HRTs, treatment plants, and seasons. Removal efficiencies increased with longer HRTs, with peak removal efficiencies reaching approximately 69%, 67%, and 61% for Na, K, and Ca, respectively. These alkali metals exhibited similar removal mechanisms, including sedimentation, filtration, chemical precipitation, adsorption, microbial interactions, and uptake by vegetation, all of which contribute to nutrient loss.

Finally, Figure 7, Figure 8, Figure 9 and Figure 10 showcase the removal efficiencies of heavy metals during the experiments at 7, 14, and 21 days HRT under CWs planted with *Phragmites karka* and *Typha latifola* across three different seasons. The figures demonstrate rapid removal of heavy metals from the wastewater, even within the 14 day treatment period. This efficient removal can be attributed to the hyperaccumulation effect of treatment plants within the CWs and various processes such as chemical precipitation, adsorption, membrane filtration, and ion exchange within the CW system. These results are consistent with findings from several studies that have reported similar patterns in heavy metal removal efficiencies from diverse wastewater sources [32,33]. Importantly, the current study noted that seasonal variations did not significantly impact the concentration levels of the detected heavy metals. Additionally, both macrophyte species remained in good condition even after a 21-day retention period, suggesting that higher removal efficiencies could be achieved with longer retention periods. It is noteworthy that, after 21 days, all detected microbial loads had been successfully degraded to non-detectable levels (ND). The tendency of the treatment plants to remove the heavy metals can be attributed to their absorption mechanisms. Polińska et al. [32] and Ansari et al. [33] reported that macrophyte plants take up pollutants/contaminants in wastewater through their roots and subsequently translocate them to their shoots. The microbial community population in the root system could aid the growth of the macrophytes, which might contribute to the enhanced phytoremediation capability in reducing the heavy metals, as retention time increases. In addition, the filtration mechanism of the constructed wetland also contributed to the reclamation of the wastewater.

The means separation between the two plants on the removal efficiency revealed that no significant difference (*p* > 0.05) was observed between the plants on the physicochemical and toxic element removal efficiency using the CW (Table S4)

### 3.2. Main and Interactive Effect of Design and Operation Factors on the Wastewater’s Physical and Chemical Properties of the VSSF-CWs

In the realm of Constructed Wetland (CW) design and operation, numerous factors require meticulous optimization to enhance the performance of these systems. Among these considerations are the choice of suitable plant species for the site, the selection of appropriate substrates, the type of wastewater being treated, the quality of plant materials used, the hydraulic loading rate (HLR), the hydraulic retention time (HRT), the prevailing climatic season, water depth, and adherence to maintenance protocols. These facets are underscored in [34,35] as crucial elements requiring optimization for CWs to achieve heightened efficiency.

In our current study, we undertook an investigation of three key factors: vegetation type, seasonal variations, and hydraulic retention time (HRT). The primary goal was to unravel both the individual and synergistic significance of these factors in shaping CW performance. To delve into the influence of these variables, we conducted comprehensive one-way and two-way Analysis of Variance (ANOVA) tests. These analyses were instrumental in unveiling the primary and interactive effects of key design and operational parameters, namely HRT, seasonal variations, and the choice of treatment plants (TPs), on the physical and chemical properties of the treated wastewater. The results of these analyses are meticulously presented in Table 4 and Table 5. Importantly, the statistical analyses were conducted at four different levels of significance, meticulously designed to mitigate the potential for type 1 and type 2 errors associated with F-tests.

Table 3 presents the findings concerning the physical, chemical, and alkali metal parameters derived from extensive experimentation on water samples. These outcomes offer compelling insights. Notably, HRT and TPs exhibited substantial effects on all the parameters studied, except for temperature. This underscores the pivotal roles played by the choice of TPs and HRT in influencing the overall efficiency of CWs. Furthermore, the influence of seasonal variations was demonstrated as significant for all tested parameters, except for turbidity and Chemical Oxygen Demand (COD). It is a well-established fact that CW performance is profoundly influenced by a plethora of factors, including the climatic region, various design considerations, and operational parameters [36]. Building upon previous research, our study yielded results that echoed some of these findings. For instance, a recent study by [22] examined the influence of HRT and seasons on a pilot-scale horizontal subsurface flow constructed wetland (HSSF-CW) designed for rural wastewater treatment. This study found that only HRT had a statistically significant effect on the CW’s performance, while the effect of seasons, although present, did not achieve statistical significance. Similarly, a study by [37] investigating the seasonal characterization of municipal wastewater and the performance evaluation of HSSF-CW in Addis Ababa, Ethiopia, concluded that the choice of TPs significantly impacted CW performance, while seasons did not. Many other studies [7,8,38] have presented similar arguments. However, it is important to note that there is no uniform consensus regarding the impact of seasons, CW types, HRT, and TP choices on CW performance.

To further scrutinize the influence of these parameters, our study employed a unique approach by exploring their interactive effects on CW performance. The results from Table 3 indicated that the interaction between HRT and seasons did not exert a significant effect on the CW’s performance in treating all pollutants, except for Na and Ca. Table 3 also revealed that the interaction between HRT and TPs significantly impacted the CW’s performance in treating all pollutants, with the exception of temperature. Furthermore, the interactions among HRT, TPs, and seasons did not reach statistical significance (ns) regarding the performance efficiency of VSSF-CW in treating pollutants from aquaculture and poultry wastewater. These results suggest that a judicious combination of TP selection and HRT can markedly enhance VSSF-CW performance, warranting further optimization efforts. In this regard, optimization efforts may be judiciously focused on HRT and TPs, as these two factors displayed significant interactive effects on all tested pollutants in this study. Earlier research has also offered valuable insights into how TP choice and HRT can influence pollutant removal in various CW types [38,39]. For instance, Calheiros et al. [40] employed HSSF-CWs in a Portuguese leather industry to treat concentrated tannery wastewater, reporting notable pollutant removal rates under specific HRT conditions. Similarly, Kaseva and Mbuligwe [41] investigated the extraction of chromium and turbidity from wastewater in Tanzania using pilot HF-CW systems, demonstrating varying pollutant removal rates in cultivated and uncultivated CWs. Another study in Bangladesh by [42] examined a combination of vertical flow (VF), horizontal flow (HF), and VF CW for industrial wastewater treatment, emphasizing the importance of substrate media and TP choice in pollutant removal.

Table 4 provided an in-depth exploration of the main and interactive effects of HRT, seasons, and TPs on the concentrations of selected heavy metal properties in treated integrated poultry and aquaculture wastewater. The results revealed the substantial influence of both HRT and TPs on the concentrations of the tested heavy metals, while seasons mostly exhibited a significant (*p* < 0.05) effect on heavy metal concentrations. This result implies that seasonal weather conditions, particularly temperature variation during the experiment, influenced the efficiency of phytoremediation. Similar observations have been reported by other researchers [43,44]. This is because temperature has a strong impact on chemical reactions in wastewater treatment, thereby regulating both biological activity and chemical processes. However, Wang et al. [45] reported that temperatures of about 30 °C and above may affect the performance of some macrophytes. The above-stated reasons confirmed why months (NDJ and MAM) mostly had lower removal efficiency, while higher maximum air temperature, mostly between 20 and 25 °C (Figures S1–S3: Supplementary Data), contributed more to the higher removal efficiency of the toxic elements in the months (JAS). This is because the heat stress that affected performance during the dry season might have reduced during the rainy season period (JAS), and consequently enhanced their performance, as revealed in the trend plot (Figures S4–S8). Haris et al. [46] reported variation in the performance of macrophytes at different temperatures. Therefore, there is a need to further establish the optimal temperature for phytoremediation of toxic metals in wastewater for different plants. When examining interactive effects, only the interaction between HRT and TPs yielded statistically significant results for Cu and Fe, with other interactions falling into the non-significant (ns) category. These outcomes reaffirmed the pivotal roles of HRT, TPs, and substrate media in shaping CW performance. In contrast, seasons appeared to have a limited impact on the removal of most heavy metals, aligning with previous observations regarding organic pollutants and alkali metals, as shown in Table 3. It is noteworthy that earlier studies have also indicated that the removal efficiencies of Cu, Al, and Zn are primarily contingent on influent concentration levels and hydraulic loading rates [36].

On a broader note, the two plant species utilized in this study effectively reduced the concentrations of all tested pollutants in integrated poultry and aquaculture wastewater to levels well below the acceptable standards for water quality, making it suitable for both irrigation and discharge into surface water bodies. The findings from Table 3 and Table 4 underscore the pivotal significance of individual factors such as HRT, seasons, and TPs in the context of VSSF-CW performance. Additionally, the combined factor of “HRT*treatment plants” was identified as significant for all tested parameters, except for Zn, turbidity, and COD. These factors assume paramount importance in optimizing the reduction of organic compounds, metals, and heavy metals in poultry and aquaculture wastewater.

## 4. Conclusions

This study evaluated the performance efficiency of vertical subsurface constructed wetlands (VSSF-CWs) with *Phragmites karka* and *Typha latifola* in treating wastewater generated from an integrated poultry and fish pond in Abeokuta, Nigeria. The experiments spanned three seasons, covering both dry and wet periods from November 2021 to September 2022. The study aimed to assess the effectiveness of VSSF-CWs by characterizing aquaculture wastewater and measuring the removal efficiency of various pollutants after 7, 14, and 21-day HRT in the CW. The initial characterization of wastewater revealed elevated concentrations of tested parameters, often exceeding local and international discharge limits. The study found significant removal efficiencies for all parameters in CWs planted with both *Phragmites karka* and *Typha latifola*. The BOD and COD reached 85 and 90% removal efficiency, while removal efficiency of 100% was achieved for most heavy metals at 21 day retention time. The capabilities of the two treatment plants in removing chemicals and heavy metals were statistically similar (*p* > 0.05). The report highlighted the efficiency of VSSF-CW in treating poultry and aquaculture wastewater, which is attributable to the absorption mechanisms of the treatment plants and the filtration mechanism of the constructed wetland. The nitrification process was accelerated by biological mechanisms, with *Phragmites karka* and *Typha latifola* thriving in nutrient-rich environments. In conclusion, this study demonstrated the potential of VSSF-CWs with *Phragmites karka* and *Typha latifola* in effectively treating wastewater, offering promising prospects for agricultural, industrial, and environmental applications in Nigerian climatic conditions. It emphasized the importance of environmental factors and plant-microbial-soil interactions in wastewater purification processes in Nigeria. The study’s recommendations include exploring specialized media (with *Phragmites karka* and *Typha latifola)*, conducting long-term studies on phytoremediation, investigating biofilter (constructed wetland) conversion processes for heavy metal extraction, enhancing plant resistance to environmental fluctuations, and researching the effects of recycled treated wastewater on crop growth. The macrophytes used in this study are therefore recommended for testing in other areas with similar or different climatic conditions, to assess the robustness and general applicability of our findings. The outcome from this study would help water scientists and local agencies in integrating the findings from this study in formulating wastewater guidelines in Nigeria, through appropriate channels like extension workers, strategies, and policies.

## Figures and Tables

**Table 1:** Methods/instruments for wastewater characterization.

Parameter	Instruments or Test Method Used
Temperature	Thermometer (model, HI 9024 HANNA, Padova, Italy)
Turbidity	Nephelometer or turbidimeter
Total hardness (TH)	Titration using ethylenediaminetetraacetic acid (EDTA)
BOD_5_	BOD self-check measurement (208215 OxiTop^®^ IS 12, Global water a Xylem brand, Milford, OH, USA)
COD	Method 8000—reactor digestion method by a spectrophotometer
Cl^−^	Silver nitrate titration method (APHA, 2012) [22]
NO_3_^−^	Spectrophotometry (colorimetric method)
SO_4_^2−^	Gravimetric sulphate determination method (APHA, 2012) [22]
Na, K, Ca, Mg	Atomic Adsorption Spectrophotometry (AAS)
As, Cd, Cu, Cr, Co, Fe, Pb, Mn, Zn, Ni	Atomic Adsorption Spectrophotometry (AAS)

**Table 2:** Average variation in concentration levels of physical and chemical properties in wastewater pre- and post-treatment across different HRTs and seasons in VSSF CW planted with *Phragmites karka* and *Typha latifolia*.

		7		14		21	
Parameters	RWW	PT	TT	PT	TT	PT	TT
Temperature (°C)	26.37 ± 0.50a	26.43 ± 0.53a	26.38 ± 0.47a	26.76 ± 0.21a	26.52 ± 0.42a	26.16 ± 0.19a	26.06 ± 0.04a
Colour (HU)	11.77 ± 0.93a	8.49 ± 0.61b	8.46 ± 0.63b	7.21 ± 0.74b	7.28 ± 0.74b	6.17 ± 0.64b	6.29 ± 0.71b
pH	8.22 ± 0.11a	7.70 ± 0.18b	7.80 ± 0.13b	7.45 ± 0.15b	7.45 ± 0.24b	7.24 ± 0.07b	7.23 ± 0.13b
DO (mg/L)	0.17 ± 0.07a	2.18 ± 0.06b	2.21 ± 0.07b	3.44 ± 0.26b	3.45 ± 0.26b	4.75 ± 0.23b	4.80 ± 0.12b
Turbidity	11.5 ± 81.19a	8.09 ± 0.45b	7.99 ± 0.43b	6.43 ± 0.59b	6.61 ± 0.50b	4.59 ± 0.48b	4.71 ± 0.61b
Acidity	123.67 ± 8.08a	92.89 ± 2.70b	93.04 ± 2.00b	76.96 ± 3.24b	73.22 ± 0.76b	60.85 ± 3.18b	60.99 ± 2.73b
Total acidity (TA)	184.33 ± 14.19a	130.00 ± 11.57b	129.58 ± 9.49b	95.770 ± 8.57b	100.36 ± 11.55b	80.04 ± 6.01b	78.04 ± 10.23b
Total hardness (TH)	74.83 ± 4.25a	59.59 ± 1.94a	59.22.77 ± 1.85b	53.23 ± 1.31b	53.55 ± 1.78b	46.15 ± 1.04b	46.68 ± 1.39b
Cl^−^	53.67 ± 6.17a	33.84 ± 3.57b	33.45 ± 2.78b	26.35 ± 1.01b	26.92 ± 1.67b	18.64 ± 1.19b	18.39 ± 0.17b
NO_3_^−^	40.17 ± 1.75a	29.00 ± 1.74b	28.77 ± 0.96b	20.00 ± 1.55b	19.62 ± 1.35b	10.21 ± 0.30b	9.50 ± 0.61b
SO_4_^2−^	117.80 ± 2.41a	78.67 ± 3.57b	77.62 ± 3.32b	57.13 ± 12.66b	55.79 ± 12.60b	33.24 ± 3.80b	34.13 ± 3.86b
BOD_5_	29.50 ± 2.27a	17.65 ± 0.63b	17.29 ± 1.54b	9.26 ± 0.43b	9.08 ± 0.32b	3.09 ± 0.52b	3.12 ± 1.25b
COD	56.67 ± 4.22a	31.73 ± 0.51b	30.49 ± 0.48b	17.77 ± 1.17b	11.79 ± 9.02b	5.79 ± 1.01b	4.97 ± 0.88b
Na	70.65 ± 6.12a	52.71 ± 9.99b	51.71 ± 10.01b	38.11 ± 5.47b	37.17 ± 5.10b	24.22 ± 2.39b	23.95 ± 0.45b
K	73.19 ± 14.71a	57.75 ± 12.26b	56.98 ± 11.91b	43.90 ± 5.16b	41.49 ± 3.41b	28.96 ± 2.40b	25.32 ± 0.79b
Ca	35.41 ± 6.73a	27.18 ± 7.27b	27.30 ± 5.64b	21.03 ± 3.83b	21.02 ± 3.05b	14.52 ± 3.20b	14.81 ± 2.94b
Mg	42.80 ± 3.57a	32.04 ± 4.43b	30.65 ± 5.11b	23.01 ± 4.86b	22.67 ± 5.91b	14.77 ± 4.41b	13.86 ± 4.86b
As	0.11 ± 0.01a	0.08 ± 0.01b	0.07 ± 0.01b	0.05 ± 0.01b	0.03b	ND	ND
Cd	0.14 ± 0.04a	0.10 ± 0.02b	0.10 ± 0.02b	0.03 ± 0.03b	0.06b	ND	ND
Cu	1.78 ± 0.17a	1.16 ± 0.25b	1.16 ± 0.27b	0.74 ± 0.28b	0.67 ± 0.30b	0.22 ± 0.05b	0.19 ± 0.03b
Cr	0.24 ± 0.04a	0.16 ± 0.02b	0.15 ± 0.05b	0.08 ± 0.02b	0.08 ± 0.02b	ND	ND
Co	0.19 ± 0.02a	0.11 ± 0.01b	0.14 ± 0.05b	0.07 ± 0.01b	0.06 ± 0.02b	ND	ND
Fe	0.84 ± 0.09a	0.41 ± 0.09b	0.42 ± 0.12b	0.22 ± 0.03b	0.23 ± 0.04b	0.13 ± 0.01b	0.14 ± 0.04b
Pb	0.21 ± 0.02a	0.13 ± 0.01b	0.13 ± 0.01b	0.07 ± 0.01b	0.07 ± 0.01b	ND	ND
Mn	0.18 ± 0.02a	0.13 ± 0.02b	0.13 ± 0.02b	0.08b	0.07 ± 0.01b	ND	ND
Zn	2.97 ± 0.12a	2.28 ± 0.11b	2.24 ± 0.14b	1.96 ± 0.13b	1.88 ± 0.11b	1.36 ± 0.04b	1.30 ± 0.14b
Ni	0.29 ± 0.08a	0.19 ± 0.05b	0.20 ± 0.05b	0.10 ± 0.01b	0.09 ± 0.01b	ND	ND

Note: Means with different letters in the row are significantly different (*p* < 0.05) for each retention time. ND means not determined.

**Table 3:** Variation in air temperature in the study area during the operation period of the VSSF CW system.

Year 2021–2022	Nov	Dec	Jan	Feb	Mar	Apr	May	Jun	Jul	Aug	Sep	Oct
T max °C	29.36	30.22	29.76	31.13	30.54	29.13	29.44	28.18	27.81	27.67	27.91	29.07
T min °C	23.70	21.66	19.54	21.59	24.38	23.80	23.79	23.02	22.42	21.67	22.54	23.11
T avg °C	26.53	25.94	24.65	26.36	27.46	26.46	26.62	25.60	25.12	24.67	25.23	26.09

**Table 4:** Main and Interactive effects of HRT (7, 14, 21 days), seasons (NDJ, MAM, JAS), and treatment plants (TPs—*Phragmites karka*, *Typha latifola*) on the physical and chemical properties of the treated integrated poultry and aquaculture wastewater.

Parameters	HRT	Seasons	TPs	HRT*Seasons	HRT*TPs	HRT*Season*TPs
	Main effect			Interactive effect		
Temperature	ns	**	ns	ns	ns	ns
Turbidity (NTU)	****	ns	****	ns	***	ns
pH	****	****	****	ns	****	ns
DO (mg/L)	****	***	****	ns	****	ns
Acidity	****	***	****	ns	****	ns
TH	****	****	****	ns	****	ns
Cl^−^ (mg/L)	****	****	****	ns	****	ns
NO_3_^−^ (mg/L)	****	**	****	ns	****	ns
SO_4_^2−^ (mg/L)	****	**	****	ns	****	ns
BOD (mg/L)	****	*	****	ns	****	ns
COD (mg/L)	****	ns	****	ns	****	ns
Na (mg/L)	****	****	****	***	****	ns
K (mg/L)	****	***	****	ns	***	ns
Ca (mg/L)	****	****	****	*	****	ns
Mg (mg/L)	***	***	****	ns	**	ns

Note: ns means non-significance; * represents significance at *p* < 0.1; ** represents significance at *p* < 0.05; *** represents significance at *p* < 0.001; **** represents significance at *p* < 0.0001.

**Table 5:** Main and Interactive effects of HRT (7, 14, 21 days), seasons (NDJ, MAM, JAS), and treatment plants (TPs—*Phragmites karka*, *Typha latifola*) on heavy-metal properties of the treated integrated poultry and aquaculture wastewater.

Parameters	HRT	Seasons	TPs	HRT*Seasons	HRT*TPs	HRT*Season*TPs
	Main effect			Interactive effect		
Cu (mg/L)	****	***	****	ns	****	ns
Fe (mg/L)	****	**	****	ns	***	ns
Zn (mg/L)	*	ns	**	ns	ns	ns

Note: ns means non-significance; * represents significance at *p* < 0.1; ** represents significance at *p* < 0.05; *** represents significance at *p* < 0.001; **** represents significance at *p* < 0.0001; HRT—Hydraulic Retention Time.

## Data Availability

The data presented in this study are available on request from the corresponding author.

## Supplementary Materials

The following supporting information can be downloaded at: https://www.mdpi.com/article/10.3390/biology14101390/s1. Table S1. Physical and chemical parameters of integrated poultry and aquaculture wastewater treated with *Phragmites karka* and *Typha latifola* in a sub-surface constructed wetland at 7, 14, and 21 day retention periods during the November 2021 to January 2022 season; Table S2. Physical and chemical parameters of integrated poultry and aquaculture wastewater treated with *Phragmites karka* and *Typha latifola* in a sub-surface constructed wetland at 7, 14, and 21 days retention periods during the March–May 2022 season; Table S3. Physical and chemical parameters of integrated poultry and aquaculture wastewater treated with *Phragmites karka* and *Typha latifola* in a sub-surface constructed wetland at 7, 14, and 21 day retention periods during the July–September 2022 season; Table S4. Mean separation for physicochemical removal efficiency in a constructed wetland as a function of seasonal variations, retention time, and treatment plants. Figure S1. Maximum air temperature trend of the study location during the experiment; Figure S2. Minimum air temperature trend of the study location during the experiment; Figure S3. Mean air temperature trend of the study location during the experiment; Figure S4. Relationship between the season of the year and Na, K, and Ca removal from the aquaculture wastewater; Figure S5. Relationship between the season of the year and Mg, As, and Cd removal from the aquaculture wastewater; Figure S6. Relationship between the season of the year and Cu, Cr, and Co removal from the aquaculture wastewater; Figure S7. Relationship between the season of the year and Fe, Pb, and Mn removal from the aquaculture wastewater; Figure S8. Relationship between the season of the year and Zn and Ni removal from the aquaculture wastewater.
