# Supplementary material for: The Effect of Retention Time and Seasonal Variation on the Characterization of Phyto-Remediated Aquaculture Wastewater in a Constructed Wetland
Source: Biology (Basel). 2025 Oct 12;14(10):1390. doi: 10.3390/biology14101390 (PMC12562205; doi:10.3390/biology14101390)
Supplement: Supplementary file 1 [file biology-14-01390-s001.zip › biology-3824967-supplementary.pdf]

Table S1: Physical and chemical parameters of integrated poultry and aquaculture wastewater treated with *Phragmites karka* and *Typha latifola* in a sub-surface constructed wetland at 7, 14- and 21- days retention periods during November 2021 to January 2022 season

**Days**		7		14		21	
Water Treatment	RWW	PT	TT	PT	TT	PT	TT
Temperature	26.90[a]	27.04[a]	26.93[a]	26.57[b]	26.40[b]	26.37[b]	26.10[c]
Colour (HU)	11.0[a]	9.16[b]	9.11[b]	7.57[b]	7.60[b]	6.90[b]	7.08[b]
Turbidity (NTU)	10.24[a]	8.49[b]	8.29[b]	6.62[b]	6.93[b]	5.10[b]	5.41[b]
pH	8.31[a]	7.82[b]	7.95[b]	7.56[b]	7.69[b]	7.30[b]	7.37[b]
DO (mg/L)	0.10[b]	2.23[a]	2.18[a]	3.22[a]	3.21[a]	4.49[b]	4.73[a]
Acidity	131.0[a]	94.67[b]	95.11[b]	79.56[b]	72.78[c]	64.33[b]	64.00[b]
TA	197.0[a]	142.67[b]	138.67[b]	11.22[b]	105.67[c]	73.89[b]	67.11[c]
TH	76.50[a]	61.42[b]	61.03[b]	54.74[b]	54.99[b]	47.03[b]	48.11[b]
Cl- (mg/L)	48.50[a]	29.92[b]	30.56[b]	25.58[b]	25.16[b]	18.04[b]	18.47[b]
NO_3^- (mg/L)	39.70[a]	30.83[b]	29.57[b]	21.56[b]	21.07[b]	10.53[b]	9.73[b]
NO_2^- (mg/L)	0.30[a]	0.093[b]	0.092[b]	ND	ND	ND	ND
SO_4^{2-} (mg/L)	115.3[a]	79.77[b]	80.08[b]	63.89[b]	62.63[b]	33.40[b]	33.84[b]
BOD (mg/L)	28.50[a]	18.37[b]	19.07[b]	9.73[b]	9.36[b]	3.29[b]	2.48[b]
COD (mg/L)	54.80[a]	31.58[b]	29.96[b]	19.02[b]	1.39[b]	5.98[b]	4.66[b]
Na (mg/L)	77.56[a]	64.02[b]	63.00[b]	44.33[b]	43.03[b]	26.87[b]	23.92[c]
K (mg/L)	81.86[a]	70.35[b]	69.90[b]	49.81[b]	45.42[b]	31.44[b]	26.22[c]
Ca (mg/L)	42.74[a]	35.45[b]	33.48[c]	25.06[b]	23.76[b]	17.04[b]	16.41[b]
Mg (mg/L)	45.90[a]	35.68[b]	35.60[b]	25.42[b]	26.90[b]	17.32[b]	17.01[b]

Note: Means that do not share a letter are significantly different; RWW means Raw Wastewater; PT stands for *Phragmites karka;* and TT stands for *Typha latifola;* ND means not detected

Table S2: Physical and chemical parameters of integrated poultry and aquaculture wastewater treated with *Phragmites karka* and *Typha latifola* in a sub-surface constructed wetland at 7, 14- and 21- days retention periods during March – May 2022 season.

Days		7		14		21	
Water treatment	RWW	PT	TT	PT	TT	PT	TT
Temperature	25.90[b]	26.14[a]	26.12[a]	26.72[a]	26.18[b]	26.12[a]	26.03[a]
Colour (HU)	12.80[a]	8.36[b]	8.42[b]	7.7[b]	7.8[b]	5.70[b]	5.70[b]
Turbidity	12.50[a]	8.17[b]	8.18[b]	6.91[b]	6.86[b]	4.54[b]	4.42[b]
pH	8.25[a]	7.79[b]	7.70[b]	7.51[b]	7.44[b]	7.26[b]	7.21[b]
DO (mg/L)	0.18[b]	2.12[a]	2.17[a]	3.36[a]	3.47[a]	4.88[a]	4.94[a]
Acidity	125.0[a]	89.78[b]	91.11[b]	78.0[b]	74.1[c]	60.1[b]	60.3[b]
TA	187.0[a]	127.33[c]	130.89[b]	100.2[c]	108.3[b]	8.33[b]	79.89[b]
TH	78.0[a]	59.8[b]	59.3[b]	52.5[b]	54.1[b]	46.41[b]	46.60[b]
Cl- (mg/L)	52.0[a]	34.7[b]	33.7[b]	27.5[b]	27.1[b]	20.01[b]	18.20[b]
NO_3^- (mg/L)	42.1[a]	28.8[b]	27.7[b]	19.97[b]	19.39[b]	10.17[b]	9.97[b]
SO_4^{2-} (mg/L)	120.1[a]	81.57[b]	78.94[c]	64.98[b]	63.49[b]	36.96[b]	38.13[b]
BOD (mg/L)	32.1[a]	17.4[b]	16.3[b]	9.14[b]	9.16[b]	3.47[b]	4.56[b]
COD (mg/L)	61.5[a]	32.3[b]	30.6[b]	17.6[b]	17.5[b]	6.69[b]	5.96[b]
Na (mg/L)	67.8[a]	45.1[b]	43.9[b]	35.96[b]	34.78[b]	23.57[b]	23.51[b]
K (mg/L)	56.73[a]	45.87[b]	46.44[b]	40.31[b]	39.37[b]	28.81[b]	24.80[c]
Ca (mg/L)	34.0[a]	24.27[b]	25.99[b]	20.61[b]	21.58[b]	15.6[b]	16.6[b]
Mg (mg/L)	43.60[a]	33.33[b]	30.95[b]	26.19[b]	25.18[b]	17.3[b]	16.3[b]

Note: Means that do not share a letter are significantly different; RWW means Raw Wastewater; PT stands for *Phragmites karka;* and TT stands for *Typha latifola;* ND means not detected

Table S3: Physical and chemical parameters of integrated poultry and aquaculture wastewater treated with *Phragmites karka* and *Typha latifola* in a sub-surface constructed wetland at 7, 14- and 21- days retention periods during July – September 2022 season.

Days		7		14		21	
Water treatment	RWW	PT	TT	PT	TT	PT	TT
Temperature	26.3[a]	26.1[a]	26.1[a]	26.99[a]	26.99[a]	25.99[b]	26.04[b]
Colour (HU)	11.50[a]	7.96[b]	7.86[b]	6.36[b]	6.43[b]	5.9[b]	6.08[b]
Turbidity (NTU)	12[a]	7.6[b]	7.5[b]	5.77[b]	6.03[b]	4.14[b]	4.3[b]
pH	8.1[a]	7.5[b]	7.75[b]	7.28[b]	7.21[b]	7.17[b]	7.12[b]
DO (mg/L)	0.23[b]	2.18[a]	2.29[a]	3.73[a]	3.72[a]	4.89[a]	4.73[a]
Acidity	115.0[a]	94.22[b]	92.89[b]	73.33[b]	72.78[b]	58.11[b]	58.67[b]
TH	70[a]	57.56[b]	57.33[b]	52.44[b]	51.56[b]	45[b]	45.33[b]
Cl- (mg/L)	60.5[a]	36.90[b]	36.10[b]	25.97[c]	28.49[b]	17.88[b]	18.50[b]
NO_3^- (mg/L)	38.70[a]	27.36[b]	29.04[b]	18.46[b]	18.41[b]	9.94[b]	8.81[b]
SO_4^{2-} (mg/L)	118[a]	74.68[b]	73.84[b]	42.53[b]	41.25[b]	29.37[b]	30.42[b]
BOD (mg/L)	27.90[a]	17.18[b]	16.51[b]	8.9[b]	8.73[b]	2.5[b]	2.33[b]
COD (mg/L)	53.70[a]	31.32[b]	30.91[b]	16.69[b]	16.49[b]	4.69[b]	4.29[b]
Na (mg/L)	65.90[a]	49.02[b]	48.24[b]	34.04[b]	33.7[b]	22.22[b]	24.41[b]
K (mg/L)	81.50[a]	57.04[b]	54.59[b]	41.59[b]	39.68[b]	26.64[b]	24.93[b]
Ca (mg/L)	29.50[a]	21.82[b]	22.43[b]	17.43[b]	17.73[b]	10.92[b]	11.42[b]
Mg (mg/L)	38.90[a]	27.10[b]	25.40[c]	17.41[a]	15.92[ab]	9.68[b]	8.26[b]

Note: Means that do not share a letter are significantly different; RWW means Raw Wastewater; PT stands for *Phragmites karka;* and TT stands for *Typha latifola;* ND

Table S4: Mean separation for physico-chemical removal efficiency in a constructed wetland as a function of seasonal variations, retention time, and treatment plants

	NDJ						MAM						JAS					
Parameters	7		14		21		7		14		21		7		14		21	
	PT	TT	PT	TT	PT	TT	PT	TT	PT	TT	PT	TT	PT	TT	PT	TT	PT	TT
Na	17.5a	18.8a	42.8a	44.5a	65.4a	69.2a	33.5a	35.3a	47.0a	48.7a	65.2a	65.3a	25.6a	26.8a	48.3a	48.9a	66.3a	63.0a
	NDJ						MAM						JAS					
	7		14		21		7		14		21		7		14		21	
	PT	TT	PT	TT	PT	TT	PT	TT	PT	TT	PT	TT	PT	TT	PT	TT	PT	TT
K	14.1a	14.6a	39.2a	44.5a	61.6a	68.0a	19.1a	18.1a	28.9a	30.6a	49.2a	56.3a	30.0a	33.0a	49.0a	51.3a	67.3a	69.4a
	NDJ						MAM						JAS					
	7		14		21		7		14		21		7		14		21	
	PT	TT	PT	TT	PT	TT	PT	TT	PT	TT	PT	TT	PT	TT	PT	TT	PT	TT
Ca	17.1a	21.7a	41.4a	44.4a	60.1a	61.6a	28.6a	23.6a	39.4a	36.5a	54.1a	51.2a	26.0a	24.0a	40.9a	39.9a	63.0a	61.3a
	NDJ						MAM						JAS					
	7		14		21		7		14		21		7		14		21	
	PT	TT	PT	TT	PT	TT	PT	TT	PT	TT	PT	TT	PT	TT	PT	TT	PT	TT
Mg	22.3a	22.4a	44.6a	41.4a	62.3a	62.9a	23.6a	29.0a	39.9a	42.2a	60.3a	62.6a	30.3a	34.7a	55.2a	59.1a	75.1a	78.8a
	NDJ						MAM						JAS					
	7		14		21		7		14		21		7		14		21	
	PT	TT	PT	TT	PT	TT	PT	TT	PT	TT	PT	TT	PT	TT	PT	TT	PT	TT
As	24.0a	26.0a	59.0b	69.0a	100.0a	100.0a	21.7b	30a	58.3b	72.5a	100.0a	100.0a	42.5a	46.7a	100.0a	100.0a	100.0a	100.0a
	NDJ						MAM						JAS					
	7		14		21		7		14		21		7		14		21	
	PT	TT	PT	TT	PT	TT	PT	TT	PT	TT	PT	TT	PT	TT	PT	TT	PT	TT
Cd	33.9a	34.4a	96.0a	66.1b	100.0a	100.0a	28.9a	25.9a	67.4a	57.0b	100.0a	100.0a	19.1a	25.5a	100.0a	100.0a	100.0a	100.0a
	NDJ						MAM						JAS					
	7		14		21		7		14		21		7		14		21	
	PT	TT	PT	TT	PT	TT	PT	TT	PT	TT	PT	TT	PT	TT	PT	TT	PT	TT

Cu	24.1a	23.0a	47.1a	48.2a	85.9a	88.5a	35.2a	37.1a	53.5a	60.4a	88.7a	88.1a	45.7a	44.6a	75.0a	78.3a	88.0a	91.3a
	NDJ						MAM						JAS					
	7		14		21		7		14		21		7		14		21	
	PT	TT	PT	TT	PT	TT	PT	TT	PT	TT	PT	TT	PT	TT	PT	TT	PT	TT
Cr	39.3a	28.6b	67.1a	69.6a	100.0a	100.0a	29.2b	37.5a	58.8a	62.5a	100.0a	100.0a	38.1b	47.6a	71.9a	74.8a	100.0a	100.0a
	NDJ						MAM						JAS					
	7		14		21		7		14		21		7		14		21	
	PT	TT	PT	TT	PT	TT	PT	TT	PT	TT	PT	TT	PT	TT	PT	TT	PT	TT
Co	40.0a	44.7a	61.8a	61.8a	100.0a	100.0a	31.3a	34.4a	54.4b	63.8a	100.0a	100.0a	51.0a	50.0a	72.5a	80.0a	100.0a	100.0a
	NDJ						MAM						JAS					
	7		14		21		7		14		21		7		14		21	
	PT	TT	PT	TT	PT	TT	PT	TT	PT	TT	PT	TT	PT	TT	PT	TT	PT	TT
Fe	37.4a	37.3a	69.1a	70.4a	84.0a	88.0a	58.5a	51.0b	76.6a	72.3a	85.1a	81.9a	55.8b	63.6a	75.3a	75.3a	83.1a	80.5a
	NDJ						MAM						JAS					
	7		14		21		7		14		21		7		14		21	
	PT	TT	PT	TT	PT	TT	PT	TT	PT	TT	PT	TT	PT	TT	PT	TT	PT	TT
Pb	38.1a	33.3a	60.5a	60.5a	100.0a	100.0a	36.8b	47.4a	60.5b	70.5a	100.0a	100.0a	39.1a	39.1a	76.1a	70.0a	100.0a	100.0a
	NDJ						MAM						JAS					
	7		14		21		7		14		21		7		14		21	
	PT	TT	PT	TT	PT	TT	PT	TT	PT	TT	PT	TT	PT	TT	PT	TT	PT	TT
Mn	32.1a	29.4a	56.1a	62.0a	100.0a	100.0a	25.0b	31.3a	51.9a	49.4a	100.0a	100.0a	25.0a	25.0a	57.0b	65.5a	100.0a	100.0a
	NDJ						MAM						JAS					
	7		14		21		7		14		21		7		14		21	
	PT	TT	PT	TT	PT	TT	PT	TT	PT	TT	PT	TT	PT	TT	PT	TT	PT	TT
Zn	21.1a	22.4a	29.9a	32.3a	52.7a	58.5a	23.9a	23.9a	35.5a	39.0a	55.8a	52.6a	24.5a	27.3a	36.7a	38.1a	54.0a	57.3a
	NDJ						MAM						JAS					
	7		14		21		7		14		21		7		14		21	
	PT	TT	PT	TT	PT	TT	PT	TT	PT	TT	PT	TT	PT	TT	PT	TT	PT	TT
Ni	36.4a	33.0a	72.6a	76.9a	100.0a	100.0a	34.6a	30.8a	64.6a	61.9a	100.0a	100.0a	34.8a	30.4a	100.0a	100.0a	100.0a	100.0a

	NDJ						MAM						JAS					
Parameters	7		14		21		7		14		21		7		14		21	
	PT	TT	PT	TT	PT	TT	PT	TT	PT	TT	PT	TT	PT	TT	PT	TT	PT	TT
Turbidity	17.1a	19.0a	35.4a	32.3a	50.2a	47.2a	34.6a	34.6a	44.7a	45.1a	63.7a	64.6a	36.7a	37.5a	52.0a	49.8a	65.5a	64.2a
	NDJ						MAM						JAS					
	7		14		21		7		14		21		7		14		21	
	PT	TT	PT	TT	PT	TT	PT	TT	PT	TT	PT	TT	PT	TT	PT	TT	PT	TT
Acidity	27.7a	27.4a	39.3a	44.4a	50.9a	51.1a	28.2a	27.1a	37.6a	40.7a	51.9a	51.8a	18.1a	19.2a	36.2a	36.7a	49.5a	48.9a
	NDJ						MAM						JAS					
	7		14		21		7		14		21		7		14		21	
	PT	TT	PT	TT	PT	TT	PT	TT	PT	TT	PT	TT	PT	TT	PT	TT	PT	TT
TA	27.6a	29.6a	40.3a	46.4a	62.5a	65.9a	31.9a	30.0a	46.4a	42.1a	55.5a	57.3a	29.0a	29.1a	49.2a	48.5a	49.2a	48.5a
	NDJ						MAM						JAS					
	7		14		21		7		14		21		7		14		21	
	PT	TT	PT	TT	PT	TT	PT	TT	PT	TT	PT	TT	PT	TT	PT	TT	PT	TT
TH	19.7a	20.2a	28.4a	28.1a	38.5a	37.1a	23.3a	24.0a	32.7a	30.6a	40.5a	40.3a	17.8a	18.1a	25.1a	26.3a	35.7a	35.9a
	NDJ						MAM						JAS					
	7		14		21		7		14		21		7		14		21	
	PT	TT	PT	TT	PT	TT	PT	TT	PT	TT	PT	TT	PT	TT	PT	TT	PT	TT
Cl-	38.3a	37.0a	47.3a	48.1a	62.8a	61.9a	33.3a	35.2a	47.1a	47.9a	61.5a	65.0a	39.0a	40.3a	57.1a	52.9a	70.4a	69.4a
	NDJ						MAM						JAS					
	7		14		21		7		14		21		7		14		21	
	PT	TT	PT	TT	PT	TT	PT	TT	PT	TT	PT	TT	PT	TT	PT	TT	PT	TT
NO_3^-	22.3a	25.5a	45.7a	46.9a	73.5a	75.5a	31.6a	34.2a	52.6a	53.9a	75.8a	76.3a	29.3a	25.0a	52.3a	52.4a	74.3a	77.2a
	NDJ						MAM						JAS					
	7		14		21		7		14		21		7		14		21	
	PT	TT	PT	TT	PT	TT	PT	TT	PT	TT	PT	TT	PT	TT	PT	TT	PT	TT
SO_4^{2-}	30.8a	30.5a	44.6a	45.7a	71.0a	70.7a	32.1a	34.3a	45.9a	47.1a	69.2a	68.3a	36.7a	37.4a	64.0a	65.0a	75.1a	74.2a
	NDJ						MAM						JAS					
	7		14		21		7		14		21		7		14		21	

	PT	TT	PT	TT	PT	TT	PT	TT	PT	TT	PT	TT	PT	TT	PT	TT	PT	TT
BOD_5	35.5a	33.1a	65.9a	67.2a	88.5a	91.3a	45.8a	49.2a	71.5a	71.5a	89.2a	85.8a	38.4a	40.8a	68.1a	68.7a	91.0a	91.7a
			NDJ							MAM						JAS		
		7		14		21		7		14		21		7		14		21
	PT	TT	PT	TT	PT	TT	PT	TT	PT	TT	PT	TT	PT	TT	PT	TT	PT	TT
COD	42.4a	45.3a	65.3a	97.5a	89.1a	91.5a	47.5a	50.2a	71.4a	71.5a	89.1a	90.3a	41.7a	42.4a	68.9a	69.3a	91.3a	92.1a

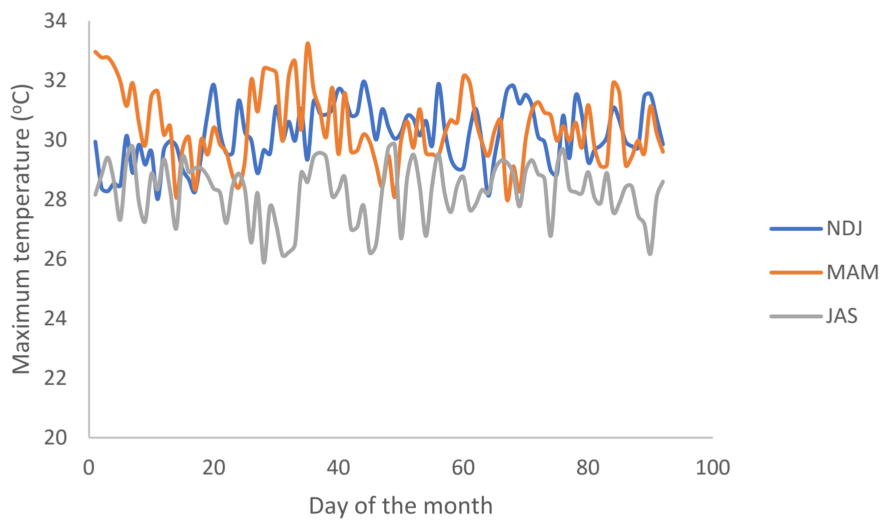

Figure S1: Maximum air temperature trend of the study location during the experiment

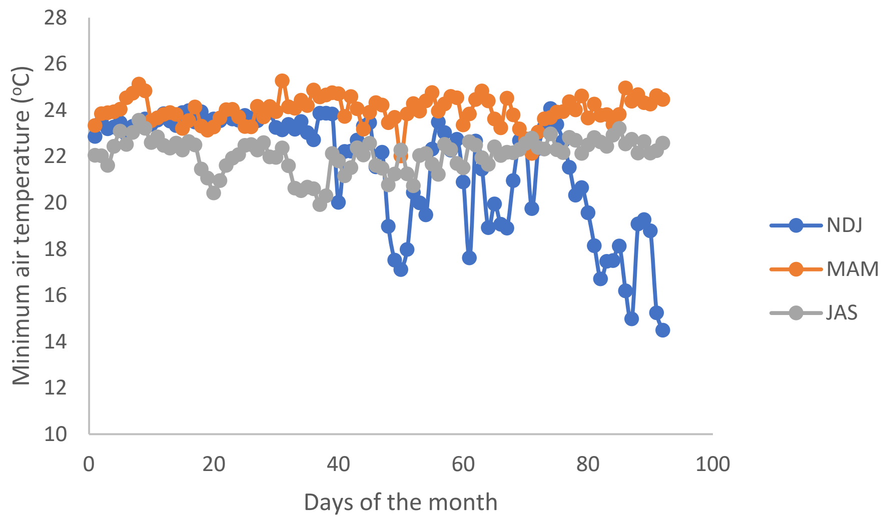

Figure S2: Minimum air temperature trend of the study location during the experiment

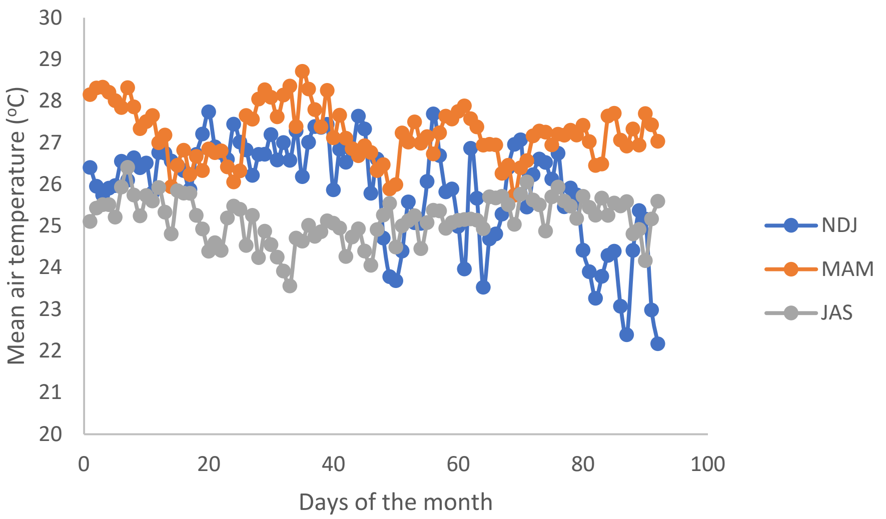

Figure S3: Mean air temperature trend of the study location during the experiment

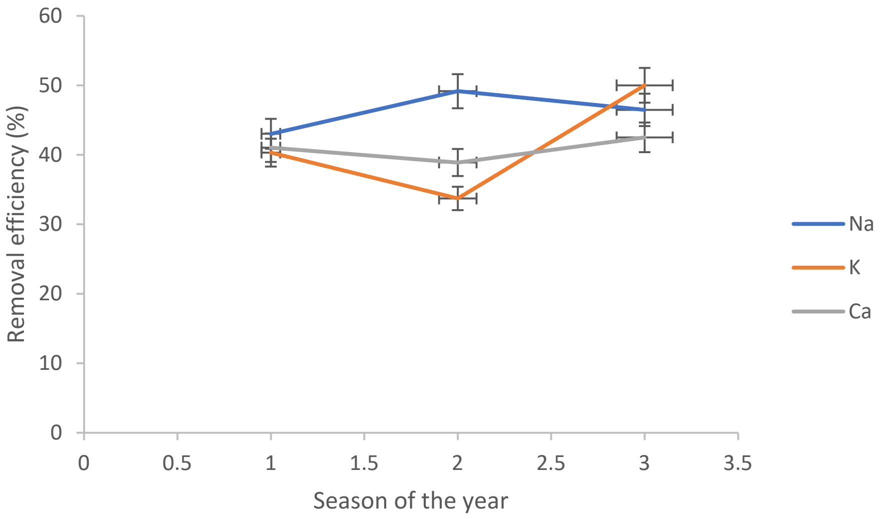

Figure S4: Relationship between the season of the year and Na, K and Ca removal from the aquaculture wastewater
Note: 1 in the horizontal axis represent NDJ, 2 represent MAM and 3 represent JAS

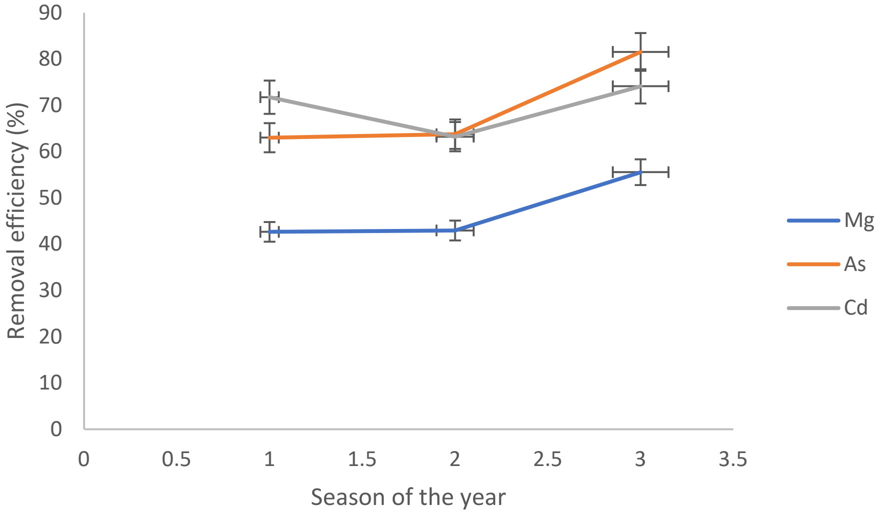

Figure S5: Relationship between the season of the year and Mg, As and Cd removal from the aquaculture wastewater

Note: 1 in the horizontal axis represent NDJ, 2 represent MAM and 3 represent JAS

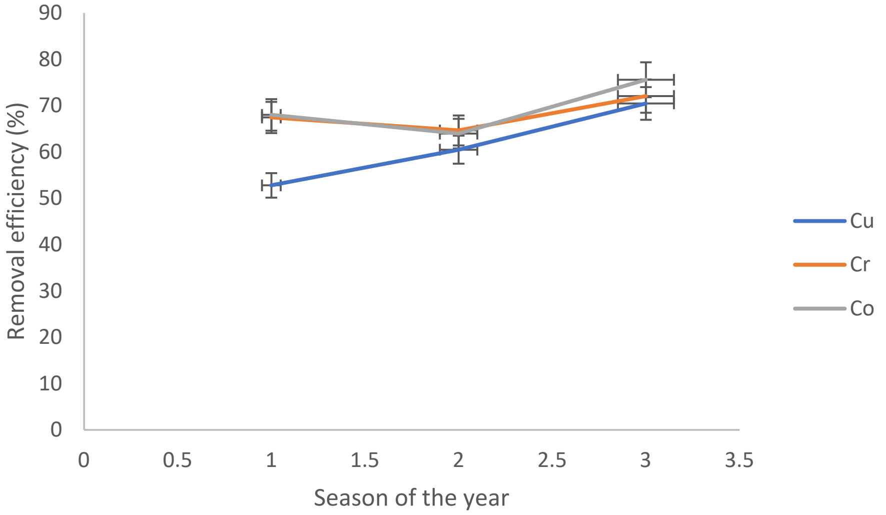

Figure S6: Relationship between the season of the year and Cu, Cr and Co removal from the aquaculture wastewater

Note: 1 in the horizontal axis represent NDJ, 2 represent MAM and 3 represent JAS

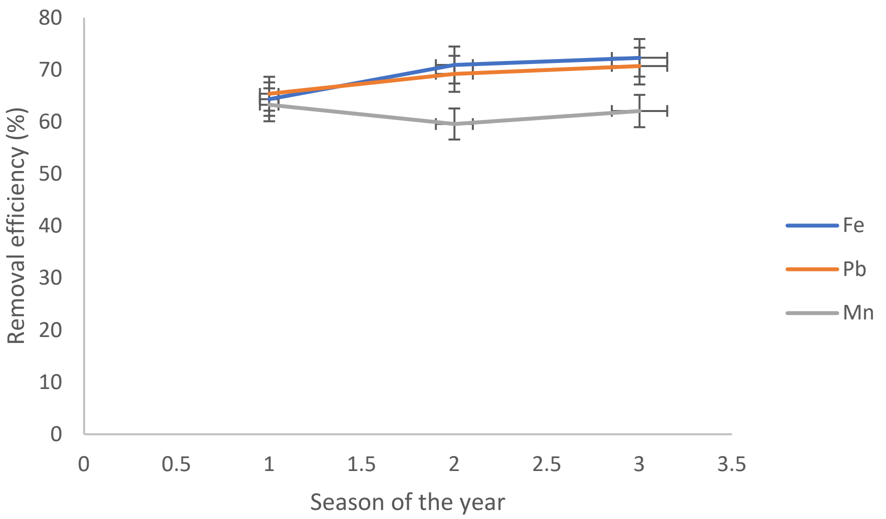

Figure S7: Relationship between the season of the year and Fe, Pb and Mn removal from the aquaculture wastewater

Note: 1 in the horizontal axis represent NDJ, 2 represent MAM and 3 represent JAS

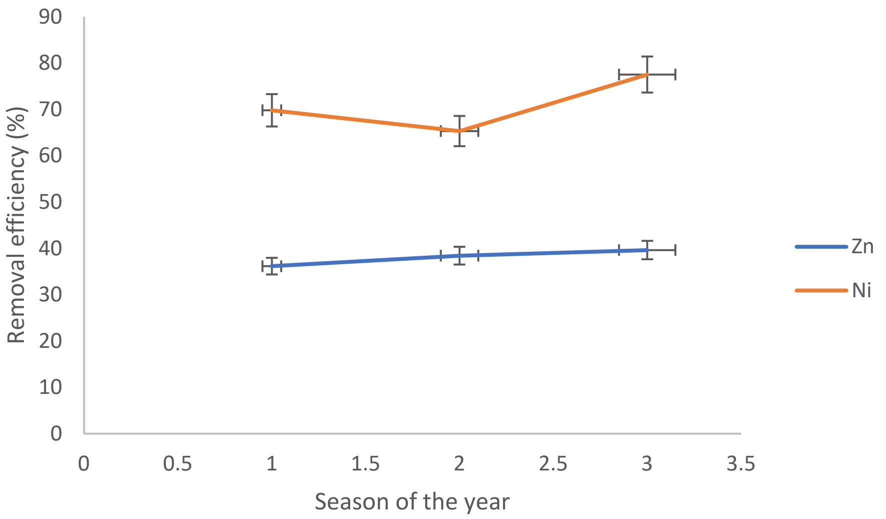

Figure S8: Relationship between the season of the year and Zn and Ni removal from the aquaculture wastewater

Note: 1 in the horizontal axis represent NDJ, 2 represent MAM and 3 represent JAS
